# Antifibrotic Mechanism of Cinobufagin in Bleomycin-Induced Pulmonary Fibrosis in Mice

**DOI:** 10.3389/fphar.2019.01021

**Published:** 2019-09-13

**Authors:** Xiaohe Li, Zhun Bi, Shuaishuai Liu, Shaoyan Gao, Yunyao Cui, Kai Huang, Mengying Huang, Jiahe Mao, Lixin Li, Jingjing Gao, Tao Sun, Honggang Zhou, Cheng Yang

**Affiliations:** ^1^State Key Laboratory of Medicinal Chemical Biology, College of Pharmacy and Tianjin Key Laboratory of Molecular Drug Research, Nankai University, Haihe Education Park, Tianjin, China; ^2^Tianjin Key Laboratory of Molecular Drug Research, Tianjin International Joint Academy of Biomedicine, Tianjin, China

**Keywords:** cinobufagin, pulmonary fibrosis, fibroblasts, epithelial-mesenchymal transition, TGF-β1

## Abstract

Idiopathic pulmonary fibrosis (IPF) is a progressive and usually fatal lung disease that is characterized by fibroblast proliferation and extracellular matrix remodeling, which result in irreversible distortion of the lung’s architecture and the formation of focal fibrous hyperplasia. The molecular mechanism by which pulmonary fibrosis develops is not fully understood, and no satisfactory treatment currently exists. However, many studies consider that aberrant activation of TGF-β1 frequently promotes epithelial-mesenchymal transition (EMT) and fibroblast activation in pulmonary fibrosis. Cinobufagin (CBG), a traditional Chinese medicine, has been widely used for long-term pain relief, cardiac stimulation, and anti-inflammatory and local anesthetic treatments. However, its role in pulmonary fibrosis has not yet been established. We investigated the hypothesis that cinobufagin plays an inhibitory role on TGF-β1 signaling using a luciferase-reporter assay. We further explored the effect of cinobufagin on pulmonary fibrosis both *in vitro* and *in vivo*. The *in vitro* experiments showed that cinobufagin suppresses TGF-β1/Smad3 signaling in a dose-dependent manner, attenuates the activation and differentiation of lung fibroblasts and inhibits EMT induced by TGF-β1 in alveolar epithelial cells. The *in vivo* experiments indicated that cinobufagin significantly alleviates bleomycin-induced collagen deposition and improves pulmonary function. Further study showed that cinobufagin could attenuate bleomycin-induced inflammation and inhibit fibroblast activation and the EMT process *in vivo*. In summary, cinobufagin attenuates bleomycin-induced pulmonary fibrosis in mice *via* suppressing inflammation, fibroblast activation and epithelial–mesenchymal transition.

## Introduction

Pulmonary fibrosis (PF) is the final stage of various interstitial lung diseases and is characterized by excessive deposition of extracellular matrix and destruction of lung parenchyma. Idiopathic pulmonary fibrosis (IPF) is the most common and ultimately fatal respiratory disease, with a mean survival of 2.5 years after diagnosis (Borensztajn et al., 2013; Maher, 2013). IPF is characterized by diffuse alveolitis and alveolar structure disorder, the formation of honeycomb focal fibrous in the patient’s lungs, and the occurrence of pulmonary interstitial fibrosis. The incidence and mortality of IPF has risen annually, which increases the medical burden (King et al., 2014). At present, there are only two drugs available for the treatment of mild/moderate IPF, pirfenidone and nintedanib, but these two drugs cannot prolong the survival of patients (Ogura et al., 2015; Jo et al., 2016). Therefore, it is important to study the pathogenesis of the disease and develop new therapeutic drugs or methods.

Although the exact etiology and pathogenesis of IPF are still unclear, it is well known that the myofibroblasts are the primary effector cell in the process of pulmonary fibrosis (Wand and Kremer, 2015). Myofibroblasts are highly produced for collagen and α-smooth muscle actin stress fibers and exhibit higher proliferation rates and a contractile phenotype. Recent evidence suggests that myofibroblasts are mainly derived from the activation/proliferation of resident fibroblasts and epithelial–mesenchymal transition (EMT) (Willis et al., 2005; Kim et al., 2006). Emerging evidence has demonstrated that EMT plays an important role in pulmonary fibrosis, while transforming growth factor-β (TGF-β) plays an important role in the EMT process. TGF-β is a key fibrogenetic factor that can activate fibroblasts that differentiate into myofibroblasts, resulting in extracellular matrix deposition (Kim et al., 2006; Hisatomi et al., 2012). Consequently, targeting the trans differentiation of myofibroblasts and TGF-β signaling may be an effective strategy in the preservation of normal lung structure during pulmonary fibrosis.

**Figure 1:**
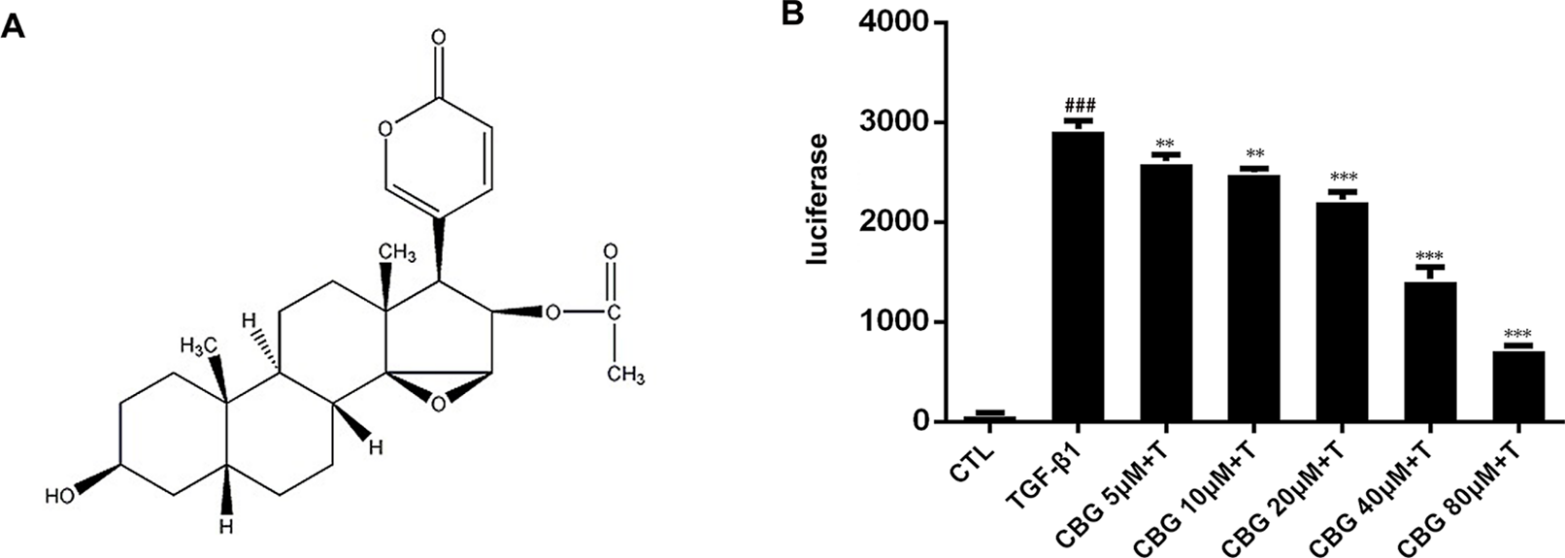
Cinobufagin inhibits TGF-β1/Smad3 signaling in fibroblasts. **(A)** Chemical structure of cinobufagin. **(B)** Cinobufagin antagonized TGF-β1-induced reporter activity in a dose-dependent manner. As described in the Materials and methods section, CAGA-NIH3T3 cells were incubated with TGF-β1 (5 ng·ml^−1^) with/without different doses of cinobufagin and then analyzed using a luciferase assay (n = 3 per group). Experiments were performed in triplicate, and statistical significance was obtained by one-way ANOVA. #represents a comparison with the blank control group, * represents a comparison with the TGF-β1 group. ^#^P < 0.05; ^##^P < 0.01; ^###^P < 0.001; P < 0.05; **P < 0.01; ***P < 0.001.

In recent years, compounds isolated from natural plants, such as Chinese medicines, have attracted the attention of researchers for their anti-fibrotic biological activities (Zhou et al., 2014; Li et al., 2015). ChanSu is made from the post-auricular and skin glands of Bufo Gargarizans (Cui et al., 2014). The main component of Chansu is cinobufagin (CBG) (**Figure 1A**), a major bioactive component of the traditional Chinese medicine, and it has been reported to have potent pharmacological activity (Zhang et al., 2007). Bufo venom contains a large number of toad toxins, which have cardiotonic activity. They are chemical steroids, and substances with such a skeleton, bufadienolide, are the main active ingredients of toad serum and toad venom. Bufotalin, cinobufagin, cinobufotalin, and bufalin are the bufadienolides in toad venom. (Li et al., 2009; Cui et al., 2010; Wang et al., 2010). In recent years, research on the pharmacological effects and clinical application of cinobufagin has confirmed that cinobufagin has anti-tumor, anti-virus, anesthetic, and analgesic effects; promotes bone marrow hyperplasia; and enhances immunity (Waiwut et al., 2012). The anti-tumor mechanisms of cinobufagin mainly include inhibiting the proliferation of tumor cells, promoting tumor cell apoptosis, reversing multi-drug resistance, and inhibiting angiogenesis and inflammation (Zhai et al., 2014; Dai et al., 2018). In 2005, cinobufagin was approved by the Chinese State Food and Drug Administration (SFDA) and then widely accepted to treat cancer patients at oncologic clinics in China. (Qi et al., 2008; Zhang et al., 2015). The role of cinobufagin in pulmonary fibrosis has not yet been established. According to the official report, murine intratracheal bleomycin model is the best-characterized animal model available for preclinical experiments of pulmonary fibrosis (Jenkins et al., 2017). Therefore, we explored the activity of cinobufagin in bleomycin-induced pulmonary fibrosis model in this study.

## Methods

### Cell Culture

Mouse fibroblast cells (NIH3T3, purchased from ATCC, passage number 32-37), (CAGA)_12_-Lux reporter stably transfected NIH 3T3 cells (CAGA-NIH3T3, passage number 30-35) and mouse lung fibroblast cells (Mlg, purchased from ATCC, passage number 25-30) were grown in DMEM (KeyGEN BioTECH, Nan Jing, China) supplemented with 10% fetal bovine serum (FBS, ExCell Bio, Shanghai, China). A549 cells (purchased from ATCC, passage number 27-29) were grown in RPMI-1640 (KeyGEN BioTECH, Nan Jing, China) supplemented with 10% FBS. Cells were maintained at 37°C with 5% CO_2_ in a humidified atmosphere.

### Animals and Bleomycin Administration

All animal care and experimental procedures complied with guidelines approved by the Institutional Animal Care and Use Committee (IACUC) of Nankai University (Permit No. SYXK 2014-0003). Animal studies are reported in compliance with the ARRIVE guidelines (Kliment and Oury, 2010; McGrath and Lilley, 2015). Six- to eight-week-old male C57BL/6 mice were obtained from Laboratory Animal Center, Academy of Military Medical Sciences of People’s Liberation Army (Beijing, China). In total, 24 mice weighing between 22 and 25 g were used in the experiments. Mice were housed under controlled temperature (22–26°C) and a 12 h light–dark cycle.

Intratracheal bleomycin administration was performed as described previously (Jiang et al., 2004). In brief, mice were anesthetized with an intraperitoneal injection of 10% chloral hydrate and then intratracheally injected with 2.5 U/kg bleomycin (Blenoxane, Nippon Kayaku Co Ltd) using a sterile insulin syringe. After injection, the mice were immediately erected and rotated left and right to make the drug solution evenly distributed in the lungs. In the sham operation group, the same amount of saline was injected intratracheally by the same method. For fibrosis model, 48 mice were divided into four groups, with 12 mice per group randomly: control group, BLM group, BLM+Pirfenidone group (200 mg·kg^−1^), and BLM+ CBG group (10 mg·kg^−1^). CBG was intragastrically administered daily for 1 week beginning 7 days after BLM injury, and Pirfenidone was used as the positive control. Control and model groups received an equal volume of vehicle (0.9% NaCl) using the same schedule and route of administration. Mice were sacrificed on the 14th day after bleomycin administration for the evaluation of pulmonary fibrosis. Six mice in each group were used for protein and mRNA analyses, and the rest of the 6 mice in each group underwent pathological analyses, with the left lung used for histology and the right lung for hydroxyproline content determination. For inflammation model, 24 mice were divided into four groups, with 6 mice per group randomly: control group, BLM group, BLM+Pirfenidone group (200 mg·kg^−1^), and BLM+ CBG group (10 mg·kg^−1^). CBG was intragastrically administered daily for 1 week beginning 1 day after BLM injury, and Pirfenidone was used as the positive control. Mice were sacrificed on the 8th day after bleomycin administration for the evaluation of inflammation.

### Pulmonary Function Testing

Mice were anesthetized with 6% chloral hydrate in saline (360 mg·kg^−1^, i.p.), tracheotomized below the larynx, and intubated with a Y-type tracheal cannula (2.5 mm inner diameter). After the surgery, the mice were placed in a supine position with their whole body inside the plethysmographic chamber (4,665 ml volume) to analyze pulmonary function using the Anires2005 system (Beijing Biolab, Beijing, China). One branch of the Y-type tracheal cannula was connected to a venthole on the chamber wall, and the venthole was connected to a ventilator from the outside of the chamber by polyethylene tubing (8 mm inner diameter, 10 cm length). Natural air was given to the animal through the ventilator, with a rate of 80 breaths/min. The other branch of the cannula was connected to the pressure-detecting channel of the Anires2005 system, and the peak inspiratory pressure was maintained at 10–16 cm H_2_O. By detecting the ventilation-associated pressure change inside the chamber, the Anires2005 system automatically calculates and displays pulmonary function parameters, such as inspiratory resistance (Ri), expiratory resistance (Re), dynamic compliance (Cdyn), forced vital capacity (FVC) and forced expiratory volume in one second (FEV1).

### Histopathological Examination

Left lungs were fixed in 10% formalin for 24 h and embedded in paraffin. Then, lung sections (4 μm) were prepared and stained with hematoxylin–eosin. Quantification of pulmonary fibrosis was performed as described previously (Jiang et al., 2004). In brief, images were photographed with an upright transmission fluorescence microscope (Olympus) and analyzed by Image-Pro Plus Version 6.0 (Media Cybernetics, Inc. American). The software selection tool can select the entire lung tissue area and automatically calculate the total pixel Pw of the region. It can then use the same method to calculate the total pixel Pf of the fibrosis region (fibrosis ratio = fibrosis area total pixel Pf/total lung total pixel Pw).

### Hydroxyproline Content Determination

The right lungs of mice were isolated, placed in 5 ml ampoules, dried, acid hydrolyzed, adjusted to pH 6.5–8.0, filtered, and adjusted to a total volume of 10 ml with 1×PBS. Each tube was comprised of 350 μl H_2_O, 50 μl sample, and 200 μl Chioramino-T. The samples were vortexed and incubated at room temperature for 20 min. Then, 200 µl perchloric acid was added, and the samples were vortexed and incubated at room temperature for 5 min. P-DMAB (200 μl) was added, and the samples were vortexed and incubated at 60°C in a water bath for 20 min. The samples were cooled, 200 μl of each sample was transferred to 96-well plates in triplicate, and the absorbance was measured at 577 nm.

### Cell Viability Analysis

NIH3T3, Mlg and A549 cells were initially seeded in 96-well plates prior to incubation for 24 h at 37°C. The cell culture media was then replaced by complete media containing the indicated concentrations (0 to 80 μM) of cinobufagin prior to incubation for specific times (24 or 48 h). After incubation as indicated, 15 μl of Thiazolyl Blue Tetrazolium Bromide (MTT, 5 mg·ml^−1^, KeyGENE BioTech, Nanjing, China) was administered to cells, followed by incubation for 4 h at 37°C following the manufacturer’s instructions. Then, 120 μl DMSO was added per well, and the absorbance was read at 570 nm by a microplate reader. Cell viability (%) was evaluated as the ratio of cells that survived.

### Luciferase Reporter Assay

For luciferase reporter assays, CAGA-NIH3T3 cells were plated in 96-well plates. When the cell density reached 80%, the DMEM containing 10% FBS was replaced with DMEM containing 1% FBS. After 24 h of incubation, cinobufagin was added, and cultures were incubated in the presence or absence of 5 ng·ml^−1^ TGF-β1 for 18 h. Cells were harvested in lysis buffer, and the luciferase activity was determined after adding the substrate.

### Wound Healing Assay

NIH 3T3 cells were seeded in a six-well plate and treated with cinobufagin in the presence or absence of TGF-β1 (5 ng·ml^−1^) for the indicated period. Three parallel lines were drawn on the underside of each well to demarcate the wound areas for analysis. Before inflicting the wound, the cells were fully confluent. A scratch was made in the center of the culture well using a sterile 200 µl micropipette tip. The wounds were observed using an inverted optical microscope, and multiple images were obtained at areas flanking the intersections of the wound and the marker lines after the scratch at 0, 6, and 24 h. Images were obtained for analysis using Image J software.

### Quantitative Real-Time PCR (qRT-PCR)

We performed RNA isolation and qRT-PCR analysis as previously described (Geng et al., 2011). The total RNA was extracted from Mlg cells and tissue using TRIzol Reagent. The cDNA was obtained from the total RNA through reverse transcription. qRT-PCR was performed using SYBR GreenER qPCR SuperMix Universal (Invitrogen) according to the manufacturer’s protocols. The relative quantification of gene expression was measured relative to the endogenous reference gene (human: β-glucuronidase; mouse: β-actin) using the comparative CT method. Sequences of the specific primer sets are as follows: α-SMA (NM_007392.2), 5-GCTGGTGATGATGCTCCCA-3 and 5-GCCCATTCCAACCATTACTCC-3; *Col1a1* (NM_007742.3), 5-CCAAGAAGACATCCCTGAAGTCA-3 and 5-TGCACGTCATCGCACACA-3; *Fn* (NM_010233.1), 5-GTGTAGCACAACTTCCAATTACGAA-3 and 5-GGAATTTCCGCCTCGAGTCT-3; *Vimentin* (NM_011701.4), 5-ATGACCGCTTTGCCAACTAC-3 and 5-GTGCCAGAGAAGCATTGTCA-3; *Cdh1 (E-cadherin)* (NM_000074.6), 5-CAGCCTTCTTTTCGGAAGACT-3 and 5-GGTAGACAGCTCCCTATGACTG-3; *IL-6* (NM_031168.2), 5’-TAGTCCTTCCTACCCCAATTTCC-3’ and 5’-TTGGTCCTTAGCCACTCCTTC-3’; *IL-1*β (NM_008361.4), 5’- GTTCCCATTAGACAACTGCACTACAG-3’ and 5’-GTCGTTGCTTGGTTCTCCTTGTA-3’; β-actin (NM_007393.3), 5-AGGCCAACCGTGAAAAGATG-3 and 5-AGAG- CATAGCCCTCGTAGATGG-3; *CDH1 (E-CAD)* (NM_000016.10), 5-CACGGTAACCGATCAGAATG-3 and 5-ACCTCCATCACAGAGGTTCC-3; *CDH2 (N-CAD)* (NM_000018.10), 5-AGCCAACCTTAACTGAGGAGT-3 and 5-GGCAAGTTGATTGGAGGGATG-3; *GUSB* (NM_000181.3), 5-CCAAACCAGCCTGACAACTT-3 and 5-TCTAGCATGCTCCACCACTG-3.

### Western Blot

The proteins were extracted from cells or lung tissues following standard protocols, as described previously (Ning et al., 2004). All of the protein was extracted from lung tissue homogenates or cells using Radio-Immunoprecipitation Assay (RIPA) lysis buffer containing phenylmethylsulfonyl fluoride (PMSF) and sodium fluoride (NaF). After electrophoresis and membrane transfer, the immunoblots were probed with the following primary antibodies: Phospho-Smad3, Smad3, α-SMA, collagen I, Fibronectin, E-cadherin, Vimentin, β-catenin, TGF-β1, ?βRI, ?βRII, and GAPDH. The secondary antibodies were goat anti-rabbit or goat anti-mouse horseradish peroxidase-conjugated antibodies. Enhanced chemiluminescence reagent was used for detection, and blots were scanned using Alphaview SA software. Three cell lines were used to analyze the above proteins: CAGA-NIH3T3 (for P-Smad3, Smad3), Mlg (for α-SMA, Collagen-1, Fibronectin), and A549 (for E-cadherin, Vimentin, β-catenin). In addition, lung tissue was used to detect all of the above proteins.

### Cell Transfection

Plasmid expressing constitutively active β-catenin (plasmid pcDNA3.1-3XHA-β-catenin) was purchased from Miao Ling Plasmid Company (China). The plasmid was transfected using Polyethylenimine, Linear (MW25000, Polysciences) according to manufacturer’s instructions. Briefly, plasmid and Polyethylenimine were diluted separately in 1 ml Opti-MEM (Gibco, Shanghai, China). The complexes were then incubated at room temperature for 20 min and added to the A549 cells. Following incubating for 4 h, the cell medium was replaced by fresh 1,640 medium with 10% FBS and the cells were incubated for further 18 h, and then stimulated with TGF-β1 and cinobufagin for 24 h.

### Immunofluorescence Staining

Cells were cultured in a 48-well chamber prior to immunofluorescence staining. After 24 h of treatment, the cells were fixed in 4% paraformaldehyde for 20 min at room temperature. After washing with PBS, cells were permeabilized with 0.2% Triton X-100 (Fluorochem) for 30 min and blocked with 5% BSA in phosphate-buffered saline (PBS) for 60 min in a humidified chamber. Mlg and A549 cells were incubated with α-SMA antibody (1:200 dilutions) and both E-cadherin (1:200 dilutions) and Vimentin (1:200 dilutions) overnight at 4°C, respectively. After washing with 1×PBS, cells were incubated with TRITC-conjugated or FITC-conjugated secondary antibody. Cell nuclei were counterstained with DAPI (Beyotime Biotechnology). Phase contrast and fluorescent microscopy was performed using an Olympus IX81 inverted research microscope.

### Immunohistochemistry Staining

Paraffin-embedded lung tissue was dewaxed with xylene, and the sections were immersed in a microwave oven with antigen-fixing solution (0.01 M citrate buffer) and retrieved after 20 min. After cooling to room temperature and blocking with 5% bovine serum albumin (BSA) for 20 min, the primary antibody was incubated at 4°C overnight. The primary antibodies were as follows: mouse anti-α-SMA antibody (1:200 dilution), rabbit anti-collagen I antibody (1:100 dilution), and rabbit anti-p-Smad3 (1:50 dilution). After washing with TBST three times, the tissue sections were incubated with the secondary antibody at 4°C at room temperature for 1 h. Subsequently, tissue sections were rehydrated through a series of ethanol solutions, and sections of lung tissues were stained with hematoxylin to observe histological changes and target gene expression in lung tissues of mice under an optical microscope.

### Bronchoalveolar Lavage

Mice were sacrificed with excessive of 10% chloral hydrate. Then, the left and right lungs were separated and underwent lavage through a blunt needle attached to a syringe, which worked as a trachea cannula in the airway. Bronchoalveolar lavage fluid (BALF) was collected by washing the lung through a tracheal intubation. The lungs were washed three times, and each time 1 ml 1XPBS was used. Total fluid recovery is about 80% per time. BALF centrifuged at 3,000 rpm for 10 min. The supernatant was kept at −80°C for following cytokine analysis, and sediment cells were resuspended with 1 ml red blood cell lysis buffer. Then the lysis buffer was centrifuged at room temperature at 3,000 rpm for 10 min, the supernatant removed and suspended again with 200 μl 1xPBS. Total cell count was performed by Countstar automatic cell counting instrument. Macrophages, neutrophils, and lymphocytes were counted under optical microscope, and 50 μl suspension was smeared and stained with HE staining.

### ELISA Detection

BALF was prepared for ELISA. The concentration of inflammatory factors including IL-1β, IL-4, IL-6, and TNF-α was detected by ELISA kits following the manufacturer’s protocol.

### Data and Statistical Analysis

The data and statistical analysis complied with the recommendations on experimental design and analysis in pharmacology (Curtis et al., 2015). Data were processed with Sigma Plot 12.5 software and expressed as the means ± SD. Kolmogorov-Smirnov tests were used to test normal distribution, and two-tailed, unpaired Student’s t tests were used to compare two groups with normal distribution. Significant differences among three or more groups were evaluated by one-way ANOVA. A value of P < 0.05 was considered statistically significant.

### Materials

Cinobufagin (CBG) was purchased from Pusi Biotechnology Co. Ltd, and the purity of CBG was 99.21%. For each experiment, CBG was freshly prepared by dissolving in DMSO (Sigma, USA). Bleomycin (BLM) was acquired from Nippon Kayaku (Tokyo, Japan). TRIzol reagent was from Ambion Life Technology. DEPC TREATED H_2_O, RNASE AWAY H_2_O and SYBR Green Real-time PCR Master Mix were from Life Technologies. M-MLV Reverse Transcriptase was purchased from Promega, and PCR Buffer without MgCl_2_ and MgCl_2_ Stock Solution were acquired from Roche. In addition, Recombinant Ribonuclease Inhibitor, dATP, dTTP, dCTP, and dGTP were acquired from Takara. RIPA lysis buffer (middle) and the BCA kit were purchased from Beyotime Biotechnology. The primary antibodies described in the study include: anti-E-cadherin, anti-Vimentin, anti-fibronectin, and anti-collagen I (Affinity Biosciences, OH, USA) and anti-GAPDH, Smad3, P-Smad3, ERK, P-ERK, JNK, P-JNK, P38, P-P38 antibody (Cell Signaling Technology, Boston, United States); α-SMA and β-catenin antibody were from Santa Cruz Biotechnology (China) and Protein-Tech (China), respectively. The secondary antibodies anti-rabbit IgG (H+L) and anti-mouse IgG (H+L) were from Applygen (Beijing). Polyethylenimine, Linear purchased from Tianjin Hao Trading. β-catenin plasmid was purchased from MiaoLingPlasmid. ELISA kits were purchased from Beijing Suobao Technology.

### Nomenclature of Targets and Ligands

Key protein targets and ligands in this article are hyperlinked to corresponding entries in http://www.guidetopharmacology.org, the common portal for data from the IUPHAR/BPS Guide to PHARMACOLOGY (Southan et al., 2016), and are permanently archived in the Concise Guide to PHARMACOLOGY 2015/16 (Alexander et al., 2015).

## Results

### Cinobufagin Inhibits TGF-β1/Smad3 Signaling in Fibroblasts

As TGF-β1 is the main promoting factor of the activation, proliferation, and differentiation of myofibroblasts, we initially assessed whether cinobufagin could inhibit the classical TGF-β1 signaling pathway. We established an NIH3T3 fibroblast cell line stably transfected with (CAGA)_12_-Lux-reporter, which encoded 12 copies of the canonical Smad3 DNA-binding sequence 5’-CAGA-3’ (the SBE). This CAGA-NIH3T3 cell line was utilized to search for compounds that could effectively inhibit the TGF-β signaling pathway (Chen et al., 2013). After the administration of 5 ng·ml^−1^ TGF-β1, CAGA-NIH3T3 cells were stimulated with different concentrations of cinobufagin (5, 10, 20, 40, and 80 μM). After 18 h, the fluorescence value of each group was detected. The results showed that cinobufagin inhibited the TGF-β1/Smad3 signaling pathway in a concentration-dependent manner (**Figure 1B**). To further study the effect of cinobufagin on TGF-β1/Smad3 signaling and pulmonary fibrosis, we performed follow-up *in vitro* and *in vivo* experiments.

### Effect of Cinobufagin on the Proliferation and Migration of TGF-β1-Activated Fibroblasts

**Figure 2:**
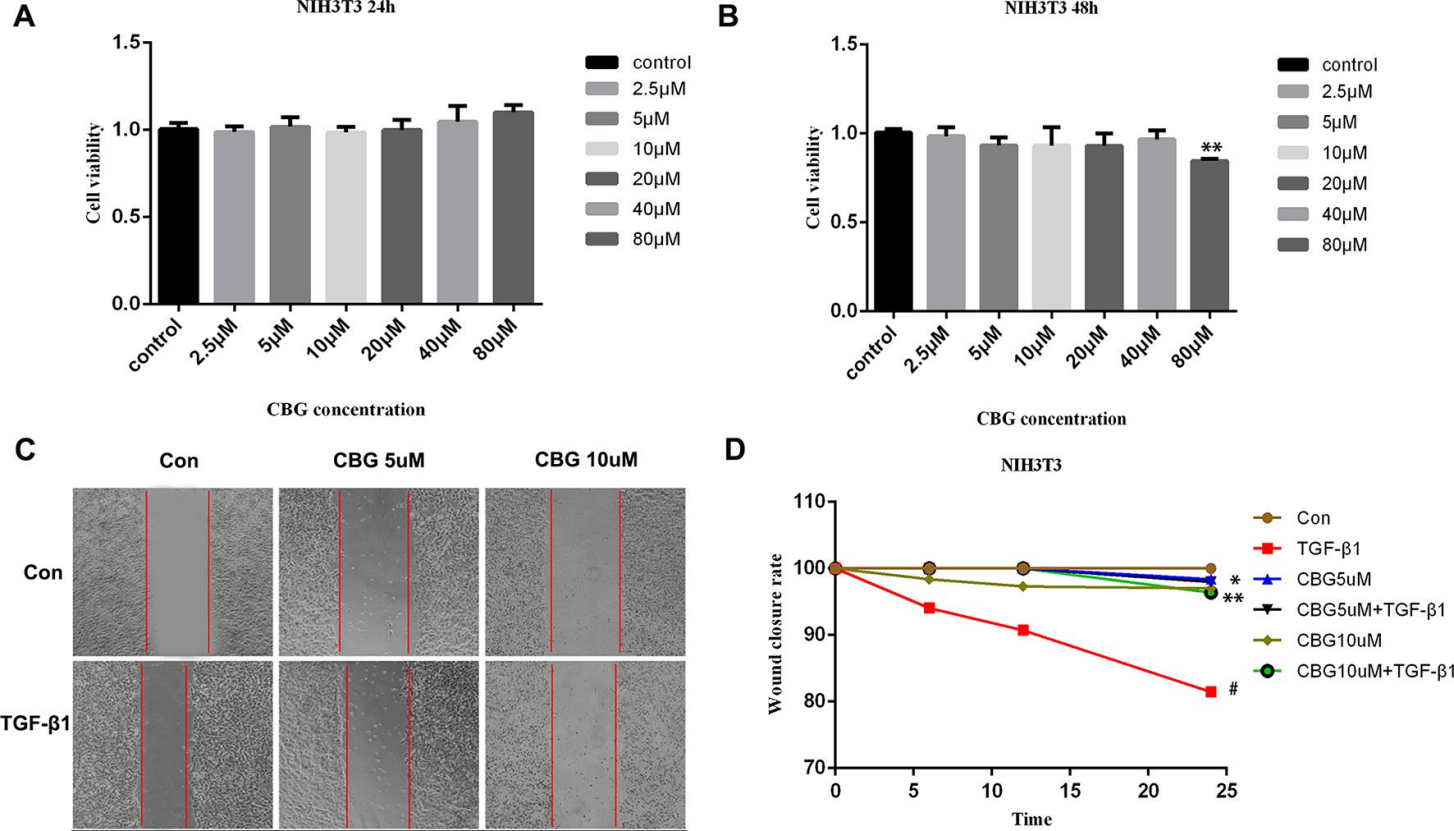
Cinobufagin inhibits TGF-β1-induced migration of lung fibroblasts. **(A, B)** NIH3T3 cells were treated with TGF-β1 (5 ng·ml^−1^) and different doses of cinobufagin for 24 h. Cells treated with DMSO served as controls. Cinobufagin was measured by MTT value-added assays at 24 and 48 h to determine the inhibitory effect of cinobufagin on fibroblast proliferation induced by TGF-β1 (n = 5 per group). **(C, D)** A wound healing assay was used to assess the effect of cinobufagin on fibroblast migration. The wound closure was photographed at 0, 12, and 24 h post-scratching (n = 3 per group). Experiments were performed in triplicate. *P < 0.05; **P < 0.01; ^#^P < 0.05.

The proliferation and migration of activated fibroblasts occur at the initial stage of tissue repair in response to injury and contribute to the development of lung fibrosis (Willis and Borok, 2007; Coward et al., 2010). To investigate the effects of cinobufagin on the proliferation and migration of fibroblasts/activated fibroblasts, NIH3T3 cells were cultured with or without 5 ng·ml^−1^ TGF-β1 and different doses of cinobufagin were administered for 24 and 48 h. We first explored whether cinobufagin affected the proliferation of NIH3T3 cells using the MTT assay. The results showed that cinobufagin did not significantly inhibit the proliferation of normal or activated fibroblasts, indicating that cinobufagin shows no cytotoxicity on fibroblasts *in vitro* (**Figures 2A, B**). Meanwhile, wound healing assays were evaluated to analyze the effects of cinobufagin on the migration of activated fibroblasts. The experimental results showed that cinobufagin inhibits the migration of activated fibroblasts (**Figures 2C, D**).

### Cinobufagin Inhibits TGF-β1-Induced Fibroblast Differentiation and ECM Production *In Vitro*

**Figure 3:**
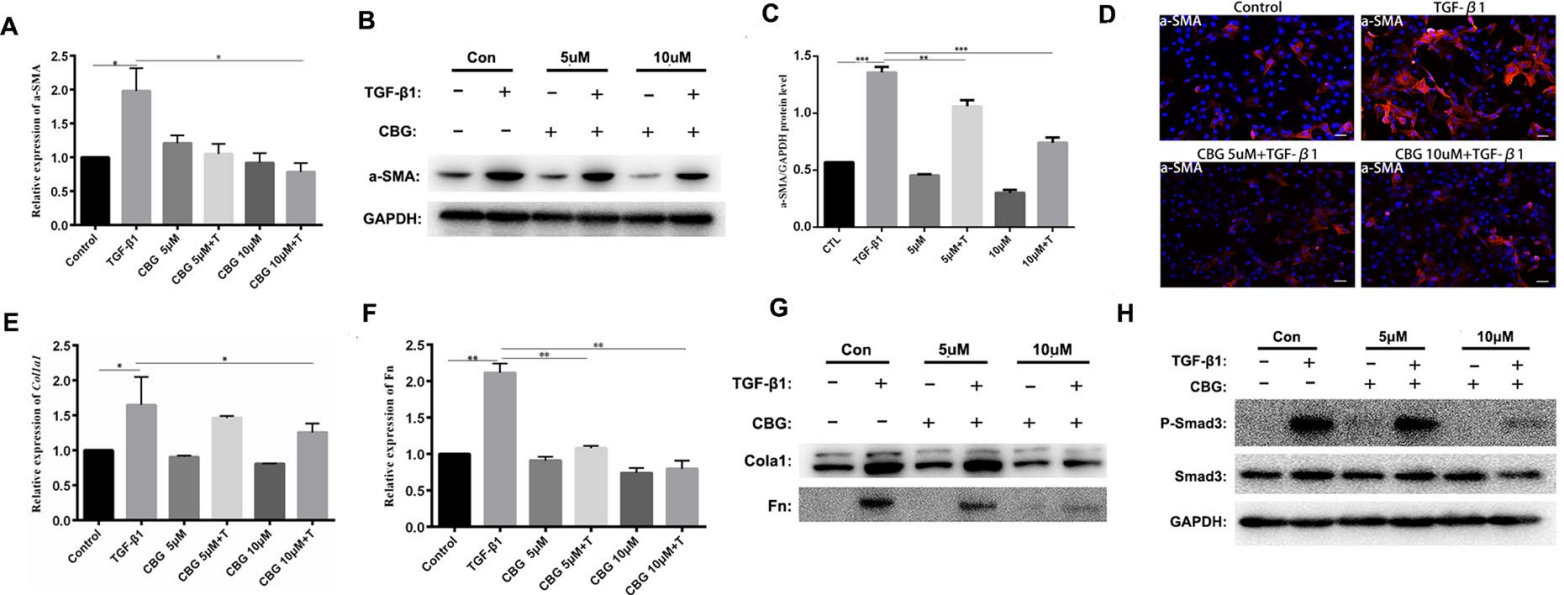
Cinobufagin inhibits TGF-β1-induced expression of α-SMA, Col1 and Fn *in vitro*. Mlg cells were treated with TGF-β1 (5 ng·ml^−1^) and cinobufagin (5 and 10 μM) for 24 h (n = 3 per group). The mRNA levels of α-SMA **(A)**, Col1a1 **(E)** and Fn **(F)** were analyzed by Real-time PCR. The protein levels of α-SMA **(B, C)**, Col1a1, Fn **(G)** and P-Smad3 **(H)** were analyzed by western blot. **(D)** Immunofluorescence staining for α-SMA (a marker of myofibroblast differentiation) was performed on Mlg cells treated with/without TGF-β1 (5 ng·ml^−1^) and/or cinobufagin (5 and 10 μM) for 24 h. *P < 0.05; **P < 0.01; ***P < 0.001.

To further investigate the inhibitory effect of cinobufagin on the TGF-β1-induced differentiation of lung fibroblasts, we treated mouse lung fibroblasts (Mlg) with TGF-β1 and different doses of cinobufagin to detect the expression of a typical activation marker: α-smooth muscle actin (α-SMA). The experimental results showed that administration of TGF-β1 substantially enhanced α-SMA mRNA expression compared with the control group, and cinobufagin (10 μΜ) treatment significantly down-regulated TGF-β1-induced α-SMA mRNA expression (**Figure 3A**). Western blotting analysis gave similar results, cinobufagin treatment significantly down-regulated TGF-β1-induced α-SMA protein expression, which indicated that cinobufagin could inhibit fibroblast differentiation into myofibroblasts (**Figures 3B, C**). We also performed immunofluorescence assays of α-SMA and obtained the same conclusion (**Figure 3D**). Extracellular matrix deposition is an important pathological feature of pulmonary fibrosis, mainly reflected in the overexpression of collagen I (Col1) and fibronectin (Fn). Not surprisingly, cinobufagin could also reduce the mRNA and protein expression levels of Col1a1 and Fn in TGF-β1-stimulated fibroblasts (**Figures 3E–G**).

In the TGF-β signaling pathway, transcription factor Smad3 plays an important regulatory role in IPF. Therefore, we examined the effect of cinobufagin on the expression and activation of Smad3. The results showed that cinobufagin could significantly reduce the phosphorylation level of Smad3 protein and had no influence on the expression of Smad3 (**Figure 3H**). Furthermore, TGF-β can also activate the MAPK pathway, in which a kinase cascade leads to the activation of ERK, JNK, and the p38 subunit. We also measured whether cinobufagin could suppress the TGF-β/MAPK signaling pathway. The results showed that cinobufagin significantly inhibited activation of ERK, JNK, and p38 without influencing the expression of MAPK (**Figure S1**). Also we found that cinobufagin had no effect on the total levels of TGF-β1 in the lungs of bleomycin treated mice and could not influence the expression levels of TGF-β receptor I and TGF-β receptor II (**Figure S2**), which indicated that cinobufagin may actualized the inhibitory role mainly through direct effect on TGF-β1 downstream signaling pathway. In summary, these data suggest that cinobufagin could inhibit fibroblast activation and reduce extracellular matrix deposition through inhibiting TGF-β1/Smad3 and TGF-β/MAPK signaling pathway.

### Cinobufagin Inhibits TGF-β1-Induced EMT in Alveolar Epithelial Cells

**Figure 4:**
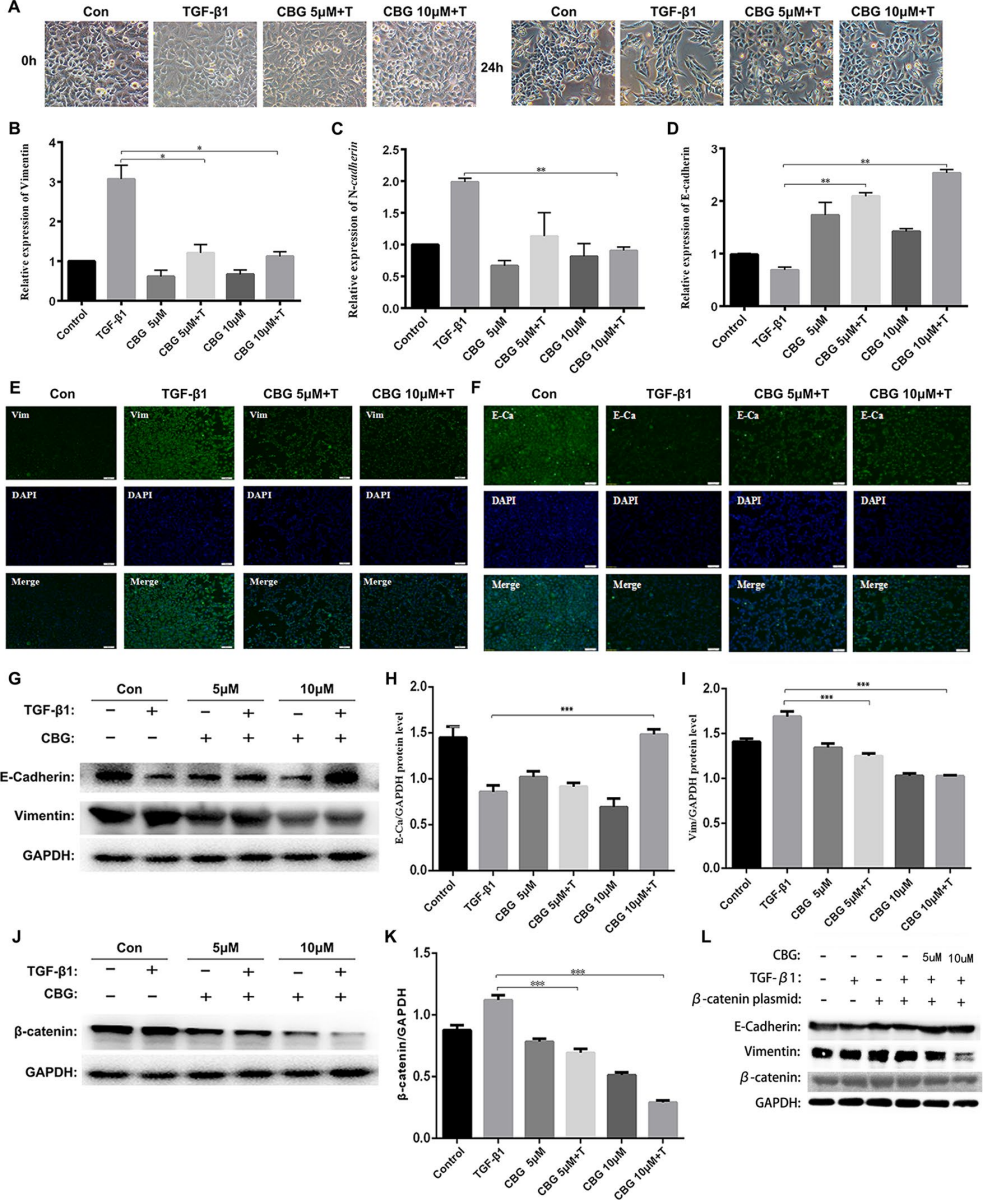
Cinobufagin suppresses TGF-β1-induced EMT in alveolar epithelial cells. A549 cells were treated with TGF-β1 and different concentrations of cinobufagin (5 and 10 µM) for 24 h. Cells stimulated with DMSO served as controls (n = 3 per group). **(A)** Morphological changes of A549 cells. **(B-D)** mRNA levels of EMT makers, including Vimentin, N-cadherin, and E-cadherin. **(E–F)** Immunofluorescence analysis of Vimentin and E-cadherin in A549 cells. **(G–I)** Quantitative densitometry of different samples by western blot. Expression levels of E-cadherin and Vimentin were normalized to GAPDH. **(J–K)** Quantitative densitometry of different samples by western blot. Expression levels of β-catenin were normalized to GAPDH. **(L)** Detection of E-Ca, Vim and β-catenin protein expression after transfection of β-catenin plasmid by Western blot. *P < 0.05; **P < 0.01; ***P < 0.001.

EMT plays an important role in pulmonary fibrosisthor (Willis and Borok, 2007). To investigate whether cinobufagin exerts anti-lung fibrosis effects by inhibiting the EMT process, we used TGF-β1 to induce EMT in A549 cells and treated cells with different doses of cinobufagin. After 24 h, the morphology changes and expression of epithelial/mesenchymal phenotypic markers of A549 cells were detected. **Figure 4A** shows that TGF-β1 resulted in extended pseudopodia and fibroblast-like morphology changes in the A549 cells, while cinobufagin treatment reversed the features of EMT to different degrees. The qRT-PCR results showed that cinobufagin can reduce the induction of mesenchymal markers by TGF-β1, such as Vimentin and N-cadherin (N–Ca), and increase the expression of the epithelial marker E-cadherin (E–Ca) (**Figures 4B–D**). Consistent with the qRT-PCR results, immunofluorescence assays showed that cinobufagin could up-regulate E–Ca expression and down-regulate Vim expression in TGF-β1-treated A549 cells (**Figures 4E, F**). Western blotting analysis showed a similar result (**Figures 4G–I**).

Since TGF-β1 signaling pathway plays an important role in the progression of EMT, and former studies showed that cinobufagin could suppressing TGF-β1/Smad3 and TGF-β/MAPK signaling pathway in the activation of fibroblasts, we further evaluated whether cinobufagin could regulated Smad3 and MAPK signaling in EMT process of A549 cells. As shown in **Figure S3**, cinobufagin could significantly inhibit TGF-β1-induced phosphorylation of Smad3 in A549 cells, but had no influence on TGF-β/MAPK signaling pathway. The results indicated different mechanisms under cinobufagin-regulated EMT and fibroblast differentiation.

Ample evidence links β-catenin activation with the induction of EMT (Kim et al., 2002; Moustakas and Heldin, 2007). Excitingly, cinobufagin also reduced the expression levels of β-catenin in TGF-β1-treated A549 cells (**Figures 4J, K**). In addition, we used the method of transfection β-catenin plasmid to verify that cinobufagin did affect EMT in pulmonary epithelial cells by regulating the expression of β-catenin. The results showed that when we overexpressed the β-catenin plasmid in A549 cells, the expression of E–Ca was down-regulated, while the expression of Vim was up-regulated. But the cinobufagin-treated group could resist the change of cells and decreased the overexpression of β-catenin (**Figure 4L**). Collectively, these data indicate that cinobufagin may inhibit the EMT process in alveolar epithelial cells by suppressing TGF-β1/Smad3 signaling and β-catenin expression.

### Cinobufagin Alleviates Bleomycin-Induced Pulmonary Fibrosis in Mice

**Figure 5:**
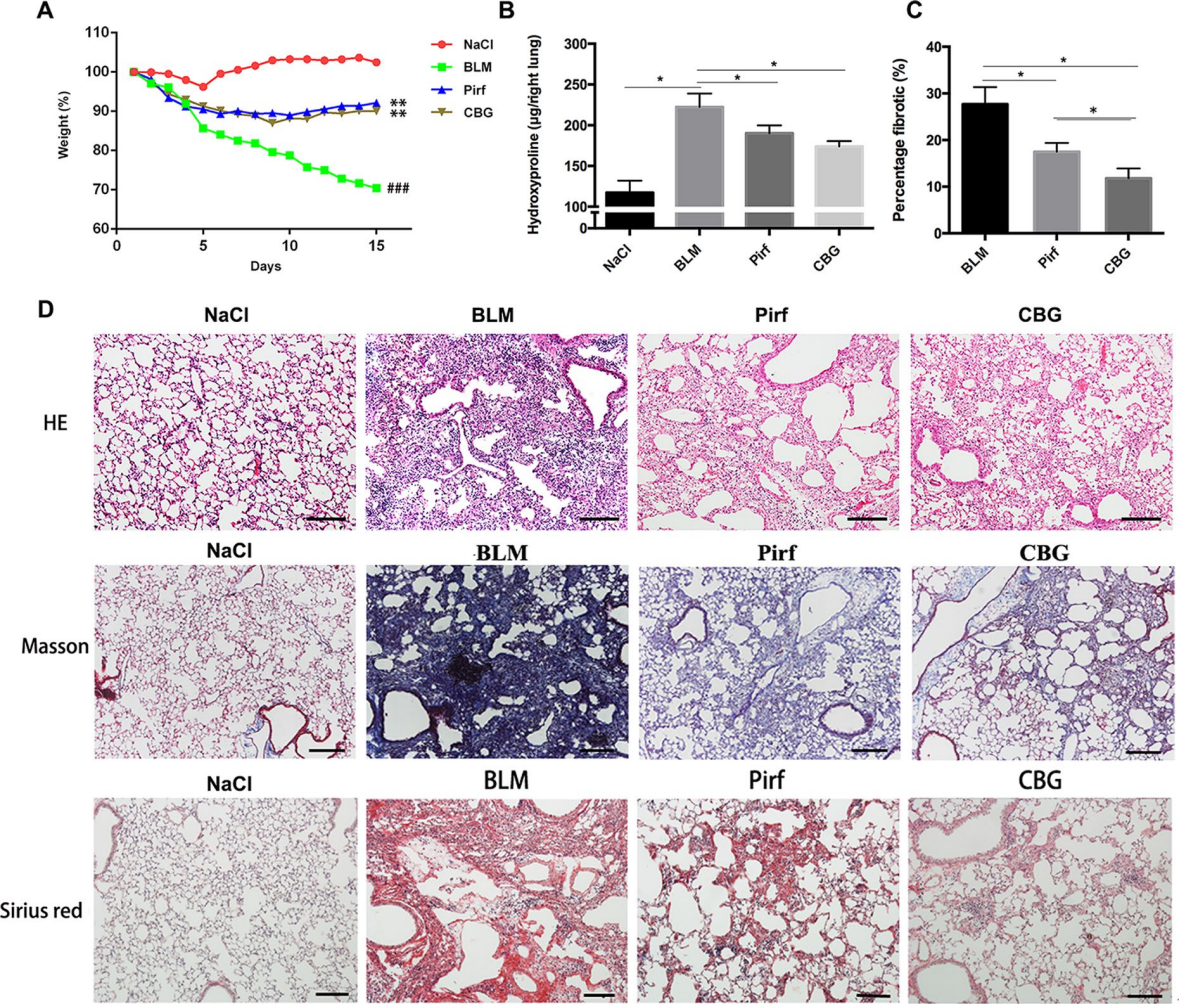
Cinobufagin attenuates BLM-induced pulmonary fibrosis in mice. Mice were intratracheally injected with a single dose of BLM (2 mg·kg^−1^). Cinobufagin (10 mg·kg^−1^) and Pirfenidone (200 mg·kg^−1^) were given from day 7 to day 14 (n = 6 per group). **(A)** Average body weight of mice in each group. **(B)** Determination of hydroxyproline content in lung tissues of each group. **(C)** Statistics of pulmonary fibrosis area in each group. **(D)** Photomicrographs of lung sections stained with hematoxylin–eosin (HE), Masson Trichrome staining and Sirius red staining. Data are presented as the means ± SD (n = 6), # represents a comparison with the NaCl control group, * represents a comparison with the BLM group. ^###^P < 0.001; *P < 0.05; **P < 0.01.

To evaluate the anti-fibrotic potential of cinobufagin *in vivo*, C57BL/6 mice were intratracheally instilled with bleomycin (BLM, 2.5U) to induce pulmonary fibrosis. On the seventh day after modeling, cinobufagin (10 mg·kg^−1^) was intraperitoneally injected and continuously administered for 7 days. Pirfenidone, a listed drug for the treatment of IPF, was used as a positive control. The results showed that the cinobufagin-administered group alleviated bleomycin-induced weight loss compared with the model group (**Figure 5A**). Treatment of bleomycin-injured mice with cinobufagin significantly reduced the collagen content and percentage of fibrotic areas compared to mice treated with Pirfenidone (**Figures 5B, C**). At the same time, HE staining, Sirius red staining and Trichrome staining were carried out to examine the histological conditions of pulmonary tissue samples. HE staining suggested that enhanced pathologic reorganization and alveolar–capillary structure damage were induced by bleomycin, while they were ameliorated by cinobufagin administration (**Figure 5D**).

**Figure 6:**
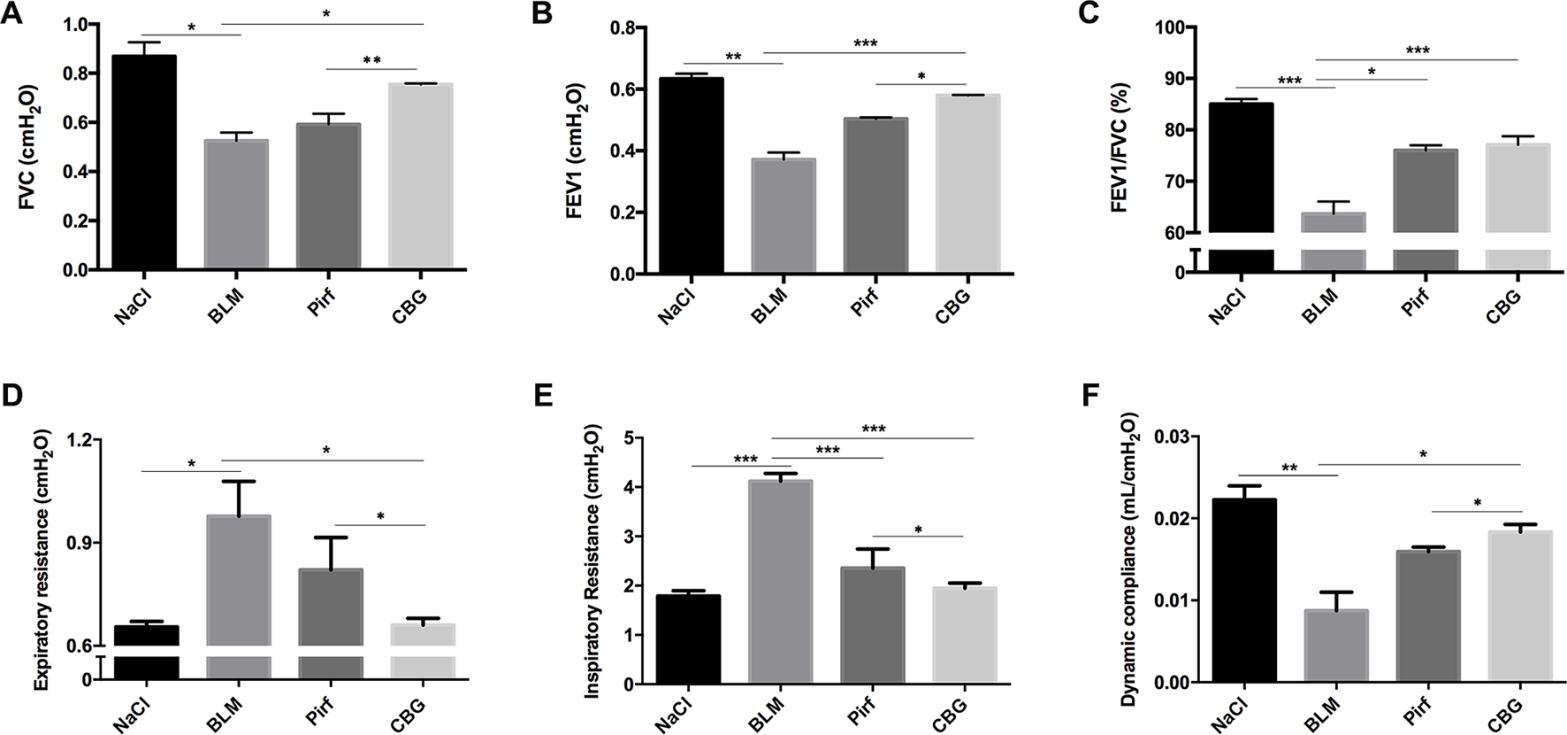
Cinobufagin improves pulmonary function in BLM-treated mice. Two weeks after BLM administration, pulmonary function of mice was determined. Pulmonary function parameters, including **(A)** Forced vital capacity (FVC), **(B)** Forced expiratory volume in one second (FEV1), **(C)** Forced expiratory volume in one second/forced expiratory volume (FEV1/FVC), **(D)** Expiratory resistance (Re), **(E)** Inspiratory resistance (Ri) and **(F)** dynamic compliance (Cdyn), were compared among different groups. Data are presented as the means ± SD (n = 6). *P < 0.05; **P < 0.01; ***P < 0.001.

We further measured the changes in pulmonary function after cinobufagin treatment. As shown in **Figure 6**, the pulmonary function of cinobufagin-treated mice was significantly improved, as seen by increased forced vital capacity (FVC), increased forced expiratory volume in one second (FEV1) and forced expiratory volume in one second/forced expiratory volume (FEV1/FVC), decreased inspiratory resistance (Ri) and expiratory resistance (Re), and increased dynamic compliance (Cdyn) when compared with bleomycin-treated mice and the Pirfenidone group. Taken together, these data suggest that cinobufagin can attenuate pulmonary fibrosis induced by BLM in mice.

### Cinobufagin Reduced the Inflammatory Response in Bleomycin-Treated Mice and Macrophage

**Figure 7:**
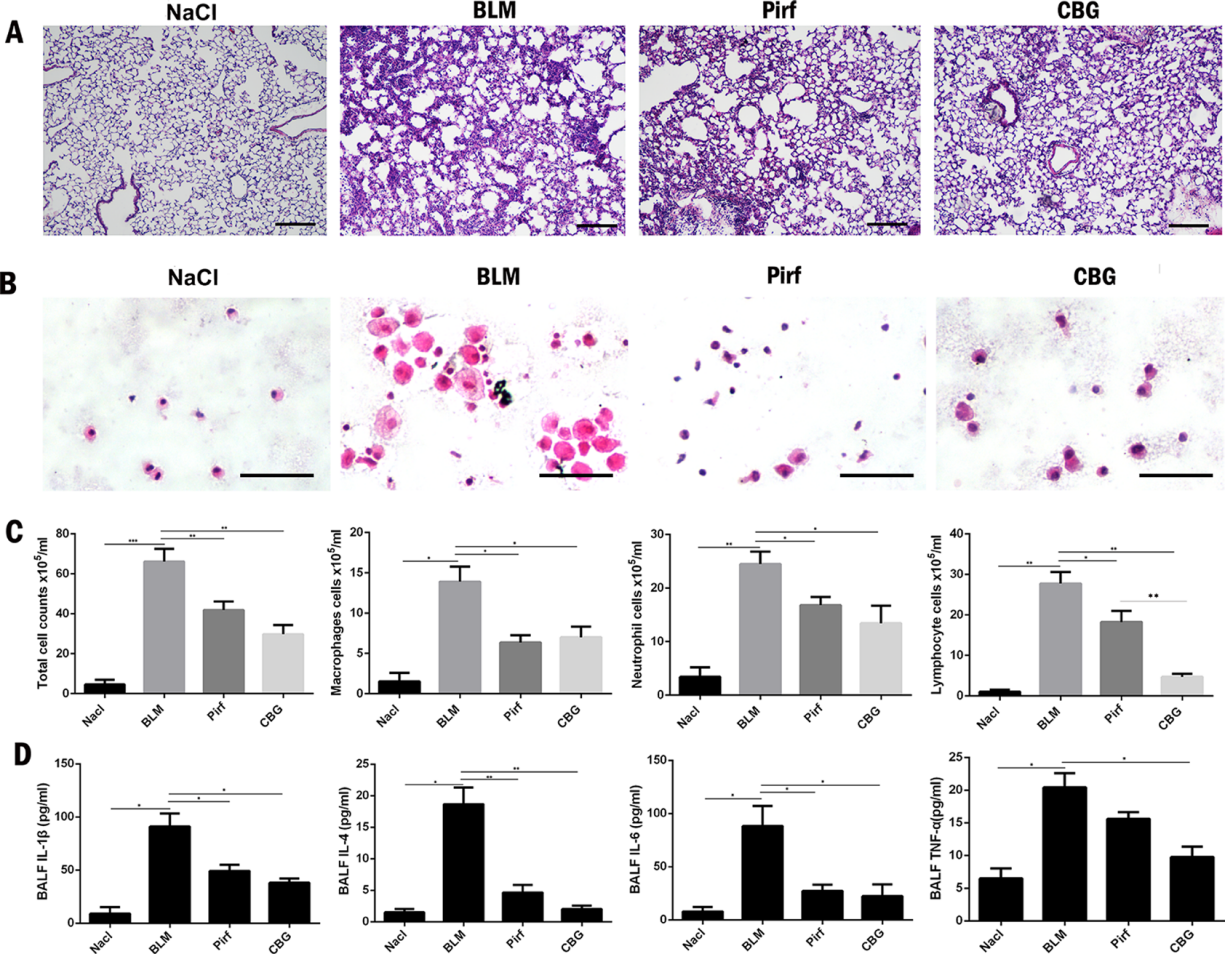
Cinobufagin reduced the inflammatory response in bleomycin-treated mice. **(A)** HE staining of left lung tissues in each group. **(B)** HE staining of inflammatory cells in the BALF. Representative visual fields of HE staining from NaCl group, BLM group, Pirfenidone group and cinobufagin group, respectively. Cells were mainly macrophages, neutrophils and lymphocytes. Original magnification, ×200. **(C)** Total cell, macrophages, neutrophils and lymphocytes from BALF in each groups were counted on day 7. **(D)** The expression of inflammatory factors including IL-1β, IL-4, IL-6 and TNF-α in BALF were detected by ELISA kit. Data are presented as mean ± SD (n = 6).*P < 0.05; **P < 0.01; ***P < 0.001.

Studies have shown that inflammation may occur in the early stage of IPF. Therefore, we used BLM intratracheal instillation to simulate the inflammatory response to mimic the early stage of IPF, and investigated whether cinobufagin could alleviate inflammatory response. HE staining of left lung pathological sections showed that cinobufagin could alleviate inflammatory cell infiltration in lung tissues, and the therapeutic effect was better than positive drug pirfenidone (**Figure 7A**). In addition, we detected the total number of cells, numbers of inflammatory cells and secretion of inflammatory cytokines including IL-1β, IL-4, IL-6, and TNF-α in BALF. It is no surprise that cinobufagin reduced the number of inflammatory cells and the expression of inflammatory factors in BALF, and the inhibitory effect was better than positive drug pirfenidone (**Figures 7B–D**). Furthermore, we detected the anti-inflammatory effect of cinobufagin *in vitro* and the results indicated that cinobufagin could inhibit the expression of IL-6 and IL-1β in LPS-treated bone marrow derived macrophages while had no effect on TGF-β1-induced expression of IL-6 and IL-1β in fibroblasts (**Figure S4**). These data indicated that cinobufagin could inhibit the inflammatory response in bleomycin-treated mice and had anti-inflammatory effect on the immune response of macrophage.

### Cinobufagin Inhibits Fibrogenic Activation of Pulmonary Fibroblasts *In Vivo*

**Figure 8:**
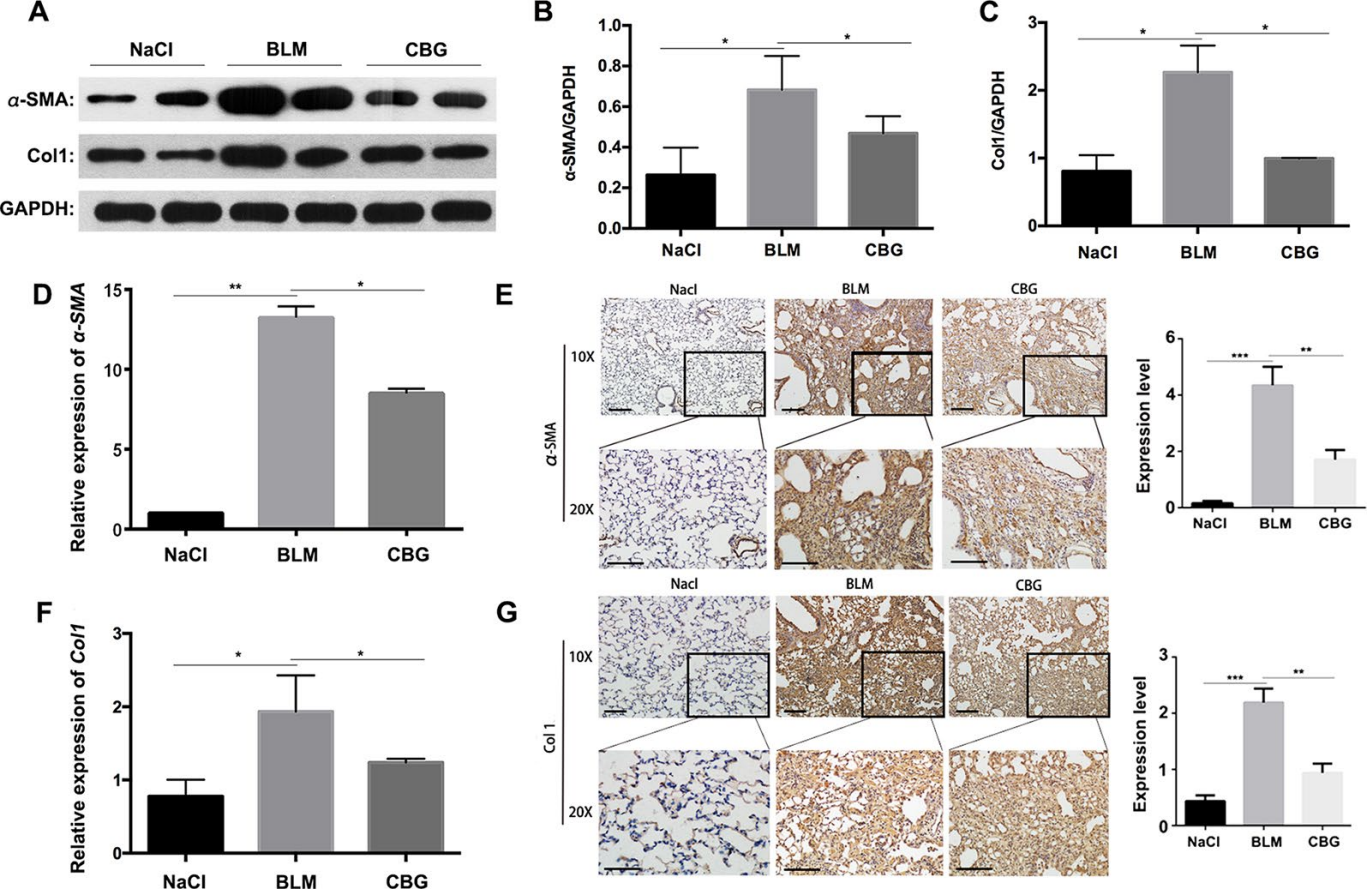
Cinobufagin attenuates BLM-induced myofibroblast differentiation and ECM deposition *in vivo*. **(A)** Western blot analysis of the protein levels of α-SMA and Col1 in lung tissues. **(B, C)** Densitometric analysis of α-SMA and Col1a1 in the immunoblots, using GAPDH as the internal reference. **(D, F)** Real-time PCR was performed to detect the mRNA levels of α-SMA and Col1a1 in the pulmonary tissues derived from each group. **(E, G)** Immunohistochemical staining of α-SMA and Col1-positive cells in the lungs. Data are expressed as the means ± SD (n = 6), *P < 0.05; **P < 0.01; ***P < 0.01.

To further verify whether cinobufagin can attenuate the activation of pulmonary fibroblasts *in vivo*, we extracted protein and RNA from the lungs of C57BL/6 mice and analyzed the expression of α-SMA and Col1 by western blotting and real-time PCR. As expected, the protein levels of and Col1 in the tissue of cinobufagin-treated lungs were lower than the bleomycin model group (**Figures 8A–C**). Similarly, in the cinobufagin-treated group, the RNA expression levels of α-SMA and Col1a1 were also significantly lower than in the bleomycin model group (**Figures 8D, F**). Subsequently, we also performed immunohistochemical studies with murine lung sections. The results also confirmed the above observation (**Figures 8E, G**). In conclusion, cinobufagin down-regulates the expression of α-SMA and Col1 in lung tissues, thereby reducing extracellular matrix deposition and releasing bleomycin-induced lung fibrosis in mice.

### Cinobufagin Prevents EMT Phenotype *In Vivo*

**Figure 9:**
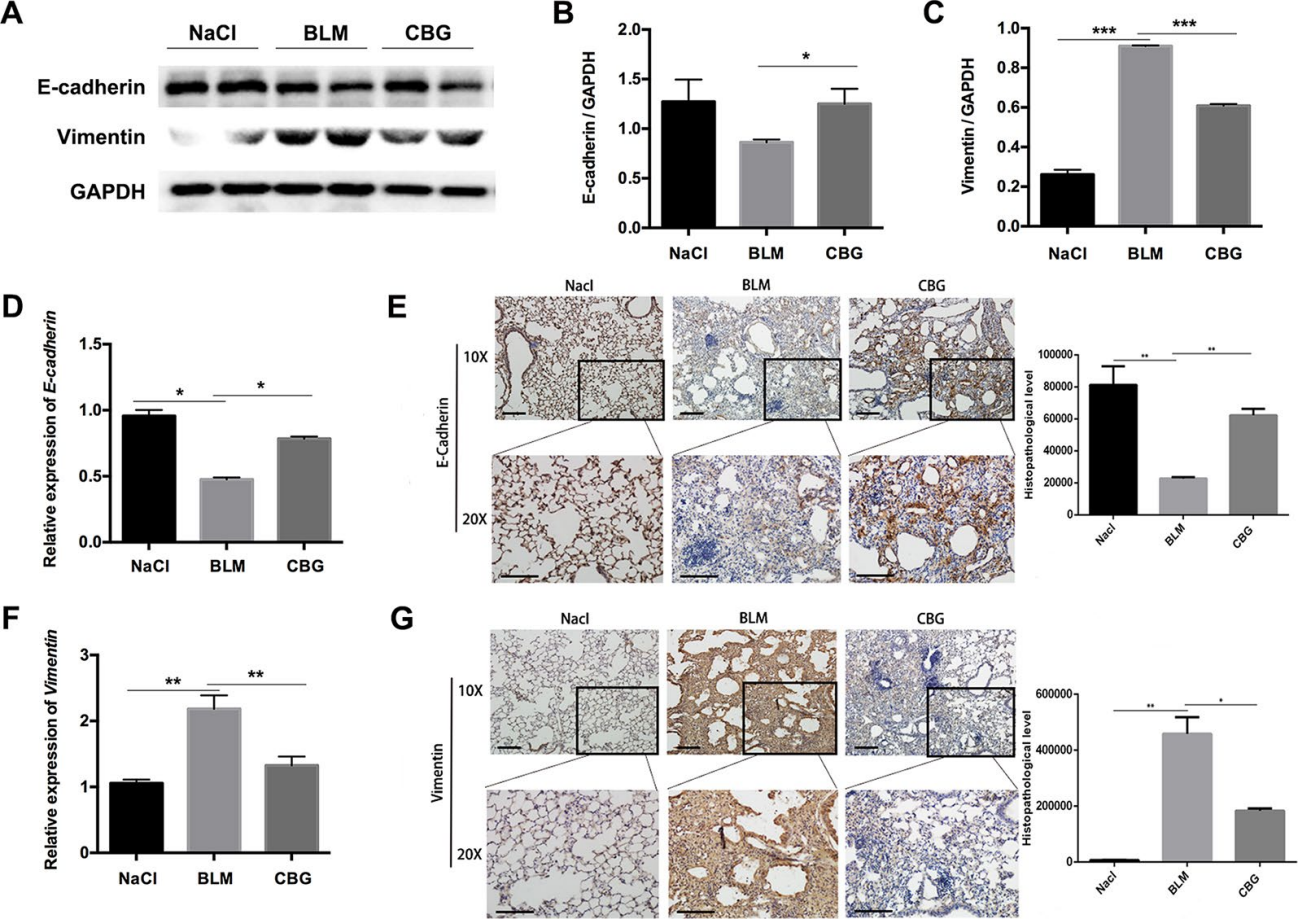
Cinobufagin represses EMT *in vivo*. **(A)** Western blot was used to analyze the protein levels of E-cadherin and Vimentin in lung tissues. **(B, C)** Densitometric analysis of E-cadherin and Vimentin in immunoblots using GAPDH as the internal reference. **(D, F)** Real-time PCR was performed to detect the mRNA levels of E-cadherin and Vimentin in the pulmonary tissues derived from each group. **(E, G)** Immunohistochemical staining of E-cadherin and Vimentin-positive cells in the lungs. Data are expressed as the means ± SD (n = 6), *P < 0.05; **P < 0.01; ***P < 0.01.

We further evaluated the role of cinobufagin in EMT progression during bleomycin-induced pulmonary fibrosis in C57BL/6 mice. Similar to our *in vitro* results, western blot assays showed that E-cadherin expression was increased and Vimentin expression decreased by cinobufagin treatment (**Figures 9A–C**). In addition, qRT-PCR analysis of lungs showed that cinobufagin up-regulates E-cadherin and down-regulates Vimentin (**Figures 9D, F**). Similarly, E-cadherin-positive cells were increased and Vimentin-positive cells reduced after cinobufagin treatment, as determined by immunohistochemical analysis (**Figures 9E, G**). All these findings provide a critical clue that cinobufagin inhibits EMT *in vivo*.

## Discussion

IPF is a chronic lung disease of unknown cause, with a progressive course, fatal prognosis and comparative morbidity and mortality. In past decades, the pathogenesis of IPF has evolved from an earlier inflammatory hypothesis to a novel hypothesis of abnormal epithelial injury repair(Strieter, 2005). IPF is characterized by an abnormal and uncontrolled proliferation of fibroblasts and myofibroblasts, distortion of lung architecture due to repetitive chronic injury to the alveolar epithelial cells, and a subsequent disorganized repair process (Sgalla et al., 2016). Injured Type II alveolar epithelial cells repeatedly release a variety of mediators, cytokines and growth factors, which promote excessive activation of fibroblasts and eventually lead to imbalances in regenerative repair and scar repair, thereby causing IPF (Selman and Pardo, 2014). Therefore, inhibiting the activation of fibroblasts is important for inhibiting the occurrence of pulmonary fibrosis.

In the present study, we investigated the possible role of cinobufagin in pulmonary fibrosis *in vitro* and *in vivo*. We firstly evaluated the inhibition role of cinobufagin on TGF-β1 signaling pathway and found that cinobufagin could inhibit the TGF-β1/Smad3 signaling in dose-dependent manner. Further data showed that cinobufagin inhibited the migration and activation of mouse fibroblasts induced by TGF-β1 *in vitro*. Intriguingly, we showed that cinobufagin had no cytotoxicity effect on fibroblasts, which is consistent with previous research (Lu et al., 2017). Thus, cinobufagin treatment was relatively safe in normal cells. *In vivo* data showed that cinobufagin effectively inhibited ECM deposition and lung remodeling and restored loss of pulmonary function. Inflammation reaction was inhibited by cinobufagin as well in bleomycin-induced lung injury. Experimental results showed that cinobufagin attenuated the expression levels of p-Smad3, α-SMA, Col1a1 and Fn, indicating that cinobufagin treatment significantly maintained the balance between the degradation of ECM components in the local lung microenvironment, which is important for the amelioration of pulmonary fibrosis.

In addition to the proliferation and differentiation of resident lung fibroblasts, myofibroblasts may also be derived, in part, from EMT. EMT, accounting for approximately 33% of myofibroblasts, has been implicated in the development of fibrotic diseases. EMT-like changes play an important role in pulmonary fibrosis. Hence, we also investigated the effects of cinobufagin on EMT in this study. In this case, we used TGF-β1 to induce EMT-like changes in human alveolar epithelial cells. We found that TGF-β1 triggered the loss of epithelial markers, such as E-cadherin, in alveolar epithelial cells and the expression of Vimentin and N-cadherin, which are specific for mesenchymal cells, consistent with previous studies (Kasai et al., 2005; Willis et al., 2005; Cheng et al., 2012; Liu et al., 2014). Cinobufagin treatment inhibited the up-regulation of TGF-β1 induced EMT *in vitro* but also suppressed EMT phenotype *in vivo*, implying that the anti-fibrotic roles of cinobufagin are, at least in part, due to EMT inhibition. Earlier studies found that β-catenin is abnormally activated in the formation of pulmonary fibrosis (Konigshoff et al., 2008; Henderson et al., 2010). Subsequently, we found that cinobufagin reduced TGF-β1-induced β-catenin overexpression. This indicates that cinobufagin may interfere with the EMT process by inhibiting β-catenin expression. Collectively, these results suggest that cinobufagin antagonizes TGF-β-mediated Smad3 and β-catenin signaling to exhibit anti-fibrotic effects. Except for the TGF-β1/Smad3 signaling pathway, we also detect whether cinobufagin could intervene the non-Smad signaling pathway (data not shown). We found that cinobufagin could only influence the Smad3 pathway and had no effect on the non-canonical pathway of TGF-β1 signaling, which indicated that cinobufagin may regulate the core effector on the TGF-β1/Smad3 signaling pathway.

However, at present, we have not studied the detailed mechanism by which cinobufagin affects TGF-β-mediated Smad3 and β-catenin signaling. Therefore, we need to study the exact effect of cinobufagin on TGF-β signaling and whether cinobufagin may also impact other signaling pathways associated with pulmonary fibrosis.

In conclusion, our results indicate that cinobufagin inhibits TGF-β1/Smad3 signaling and subsequently suppresses fibroblast activation and differentiation, EMT and eventual ECM deposition. Furthermore, cinobufagin treatment leads to improved bleomycin-induced pulmonary fibrosis and inflammatory responses in mice. Based on these observations, we believe that cinobufagin has a variety of roles other than anti-tumor effects and may be used as an anti-fibrotic drug candidate. As a traditional Chinese medicine monomer, cinobufagin has better pharmacodynamic effect on IPF than Pirfenidone. In addition to inhibiting the activation of a series of fibroblasts induced by activated TGF-beta/smad3, cinobufagin can also reduce the source of myofibroblasts by resisting EMT and further improve pulmonary fibrosis. Therefore, on the one hand, we want to develop cinobufagin as a new indication of anti-pulmonary fibrosis, and at the same time, we hope that the mother nucleus of cinobufagin (i.e. effective group) can be used as a candidate for the development of new anti-pulmonary fibrosis compounds, so as to provide more drug options for clinical IPF patients.

## Ethics Statement

All animal care and experimental procedures were complied with guidelines approved by the Institutional Animal Care and Use Committee (IACUC) of Nankai University (Permit No. SYXK 2014-0003). Animal studies are reported in compliance with the ARRIVE guidelines (Kliment and Oury, 2010; McGrath and Lilley, 2015).

## Author Contributions

CY, HZ, and TS contributed to the conception and design. XL, SL, ZB and MH contributed to the collection and assembly of data. JM, SG, and KH contributed to the data analysis and interpretation. YC contributed to the data revision. XL, ZB and SL contributed to the manuscript writing. JG and LL contributed to the administrative support. All authors read and approved the final manuscript.

## Funding

This study was supported by the National Science and Technology Major Projects for “Major New Drugs Innovation and Development” [Grant SQ2018ZX090201], Open fund project of State Key Laboratory of Medicinal Chemical Biology [Grant 2018128], and Fundamental Research Funds for the Central Universities.

## Conflict of Interest Statement

The authors declare that the research was conducted in the absence of any commercial or financial relationships that could be construed as a potential conflict of interest.

## Abbreviations

α-SMA, α-smooth muscle actin; BLM, bleomycin; Col1, collagen-1; CBG, Cinobufagin; ECM, extracellular matrix; EMT, epithelial-mesenchymal transition; Fn, fibronectin; PF, pulmonary fibrosis; IPF, idiopathic pulmonary fibrosis; TGF-β1, Transforming growth factor-β1.

## Supplementary Material

Figure S1 Cinobufagin inhibits TGF-β1/MAPK signaling in lung fibroblasts. Mlg cells were treated with TGF-β1 (5 ng·ml^−1^) and cinobufagin (5 and 10 μM) for 1 h (n = 3 per group). The protein levels of ERK, P-ERK, JNK, P-JNK, P38, and P-P38 were analyzed by western blot. GAPDH was used as a loading control.

Figure S2 Cinobufagin had no effect on the total levels of TGF-β1 and TGF-β receptor. **(A)** Western blot was used to analyze the protein levels of TGF-β1 in lung tissues. **(B)** Densitometric analysis of TGF-β1 in immunoblots using GAPDH as the internal reference. **(C)** Mlg cells were treated with TGF-β1 (5 ng·ml^−1^) and cinobufagin (5 and 10 μM) for 24 h (n = 3 per group). The protein levels of TGF-β receptor I (TβRI) and TGF-β receptor II (TβRII) were analyzed by western blot. **(D, E)** Densitometric analysis of TβRI and TβRII in immunoblots using GAPDH as the internal reference.

Figure S3 Cinobufagin inhibits TGF-β1/Smad3 signaling in alveolar epithelial cells. A549 cells were treated with TGF-β1 (5 ng·ml^−1^) and cinobufagin (5 and 10 μM) for 1 h (n = 3 per group). The protein levels of **(A)** Smad3 and P-Smad3, **(B)** ERK, P-ERK, JNK, P-JNK, P38, and P-P38 were analyzed by western blot. GAPDH was used as a loading control.

Figure S4 Cinobufagin inhibits LPS-induced expression of IL-6 and IL-1β in bone marrow derived macrophages. **(A, B)** Cultured bone marrow derived macrophages were treated with LPS (1 μg·ml^−1^) and cinobufagin (5 and 10 μM) for 12 h (n = 3 per group). The RNA levels of IL-6 **(A)** and IL-1β **(B)** were analysed by RT-PCR. **(C, D)** Mlg cells were treated with TGF-β1 (5 ng·ml^−1^) and cinobufagin (5 and 10 μM) for 12 h (n = 3 per group). The RNA levels of IL-6 **(C)** and IL-1β **(D)** were analysed by RT-PCR. Data are expressed as the means ± SD, *P < 0.05; ***P < 0.01.
