# Supplementary figures and images for: Antifibrotic Mechanism of Cinobufagin in Bleomycin-Induced Pulmonary Fibrosis in Mice
Source: Front Pharmacol. 2019 Sep 13;10:1021. doi: 10.3389/fphar.2019.01021 (PMC6753632; doi:10.3389/fphar.2019.01021)

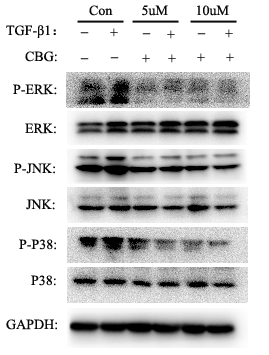

Supplement: Figure S1 — Cinobufagin inhibits TGF-β1/MAPK signaling in lung fibroblasts. Mlg cells were treated with TGF-β1 (5 ng·ml−1) and cinobufagin (5 and 10 μM) for 1 h (n = 3 per group). The protein levels of ERK, P-ERK, JNK, P-JNK, P38, and P-P38 were analyzed by western blot. GAPDH was used as a loading control. [file Image_1.tif]

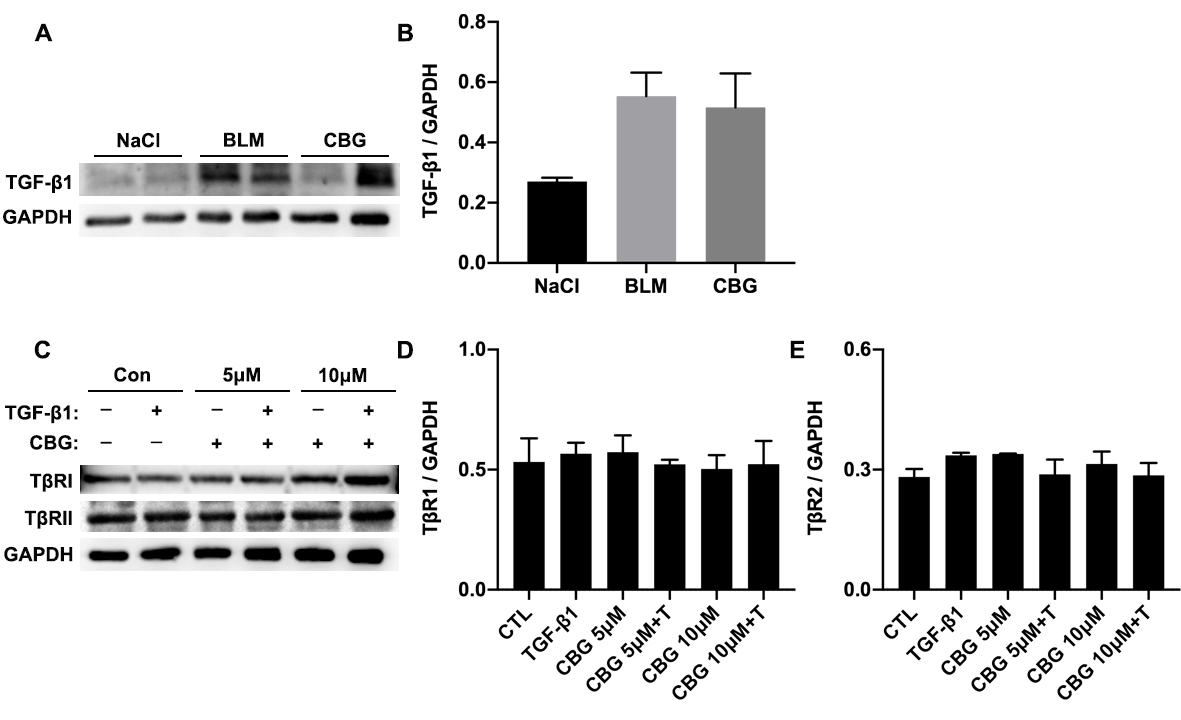

Supplement: Figure S2 — Cinobufagin had no effect on the total levels of TGF-β1 and TGF-β receptor. (A) Western blot was used to analyze the protein levels of TGF-β1 in lung tissues. (B) Densitometric analysis of TGF-β1 in immunoblots using GAPDH as the internal reference. (C) Mlg cells were treated with TGF-β1 (5 ng·ml−1) and cinobufagin (5 and 10 μM) for 24 h (n = 3 per group). The protein levels of TGF-β receptor I (TβRI) and TGF-β receptor II (TβRII) were analyzed by western blot. (D, E) Densitometric analysis of TβRI and TβRII in immunoblots using GAPDH as the internal reference. [file Image_2.tif]

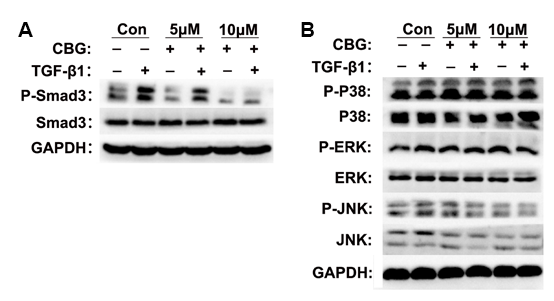

Supplement: Figure S3 — Cinobufagin inhibits TGF-β1/Smad3 signaling in alveolar epithelial cells. A549 cells were treated with TGF-β1 (5 ng·ml−1) and cinobufagin (5 and 10 μM) for 1 h (n = 3 per group). The protein levels of (A) Smad3 and P-Smad3, (B) ERK, P-ERK, JNK, P-JNK, P38, and P-P38 were analyzed by western blot. GAPDH was used as a loading control. [file Image_3.tif]

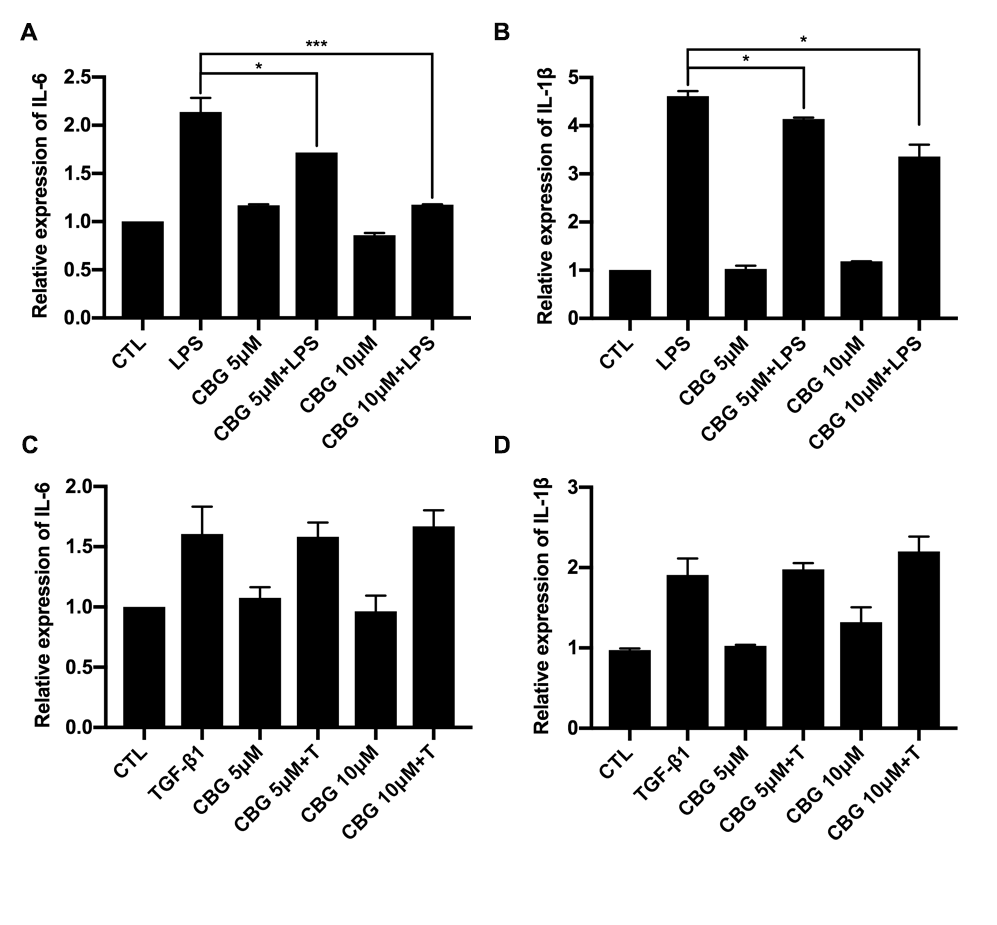

Supplement: Figure S4 — Cinobufagin inhibits LPS-induced expression of IL-6 and IL-1β in bone marrow derived macrophages. (A, B) Cultured bone marrow derived macrophages were treated with LPS (1 μg·ml−1) and cinobufagin (5 and 10 μM) for 12 h (n = 3 per group). The RNA levels of IL-6 (A) and IL-1β (B) were analysed by RT-PCR. (C, D) Mlg cells were treated with TGF-β1 (5 ng·ml−1) and cinobufagin (5 and 10 μM) for 12 h (n = 3 per group). The RNA levels of IL-6 (C) and IL-1β (D) were analysed by RT-PCR. Data are expressed as the means ± SD, *P < 0.05; ***P < 0.01. [file Image_4.tif]
